# Field Epidemiology and Laboratory Training Program, Where Is the L-Track?

**DOI:** 10.3389/fpubh.2018.00264

**Published:** 2018-09-19

**Authors:** Wangeci Gatei, Tura Galgalo, Ahmed Abade, Alden Henderson, Mark Rayfield, David McAlister, Joel M. Montgomery, Leonard F. Peruski, Adilya A. Albetkova

**Affiliations:** ^1^Division of Global Health Protection, Center for Global Health, Centers for Disease Control and Prevention, Atlanta, GA, United States; ^2^FELTP Program, African Field Epidemiology Network (AFENET) at National Health Laboratory Quality Assurance and Training Center, Dar Es Salaam, Tanzania

**Keywords:** FELTP, laboratory track, L-track, laboratory workforce, global health security

## Abstract

**Background:** Modifications of the Field Epidemiology Training Program (FETP) curricula to include a laboratory track (L-Track), to become Field Epidemiology and Laboratory Training Program (FELTP), began in 2004 in Kenya. The L-Track offered candidates training on laboratory competencies in management, policy, quality systems, and diagnostic methods as well as epidemiology, disease surveillance and outbreak response. Since then several FELTPs have discontinued the L-Track and instead offer all candidates, epidemiologists and laboratorians, a single FETP curriculum. Reasons for these changes are reported here.

**Methods:** A questionnaire was sent to directors of 13 FELTP programs collecting information on the status of the programs, reasons for any changes, basic entry qualifications, source institutions and where residents were post enrollment or after graduation. Data from previous CDC internal assessments on FELTP L-Track was also reviewed.

**Results:** Out of the 13 FELTPs included, directors from 10 FELTPs sent back information on their specific programs. The FELTPs in Kenya, Mozambique, Cameroon and Kazakhstan and Mali have discontinued a separate L-Track while those in Ghana, Georgia, Nigeria, Rwanda, and Tanzania continue to offer the separate L-Track. Reasons for discontinuation included lack of standardized curriculum, unclear strategies of the separate L-Track, and funding constraints. Two countries Kenya and Tanzania reported on the career progression of their graduates. Results show 84% (Kenya) and 51% (Tanzania) of candidates in the FELTP, L-Track were recruited from national/regional medical health laboratories. However post-graduation, 56% (Kenya) and 43% (Tanzania) were working as epidemiologists, program managers, program coordinators, or regulatory/inspection boards. Professional upward mobility was high; 87% (Kenya) and 73% (Tanzania) residents, reported promotions either in the same or in new institutions.

**Conclusions:** The FELTP L-Track residents continue to offer critical contributions to public health workforce development with high upward mobility. While this may be a reflection of professional versatility and demand of the FELTP graduates, the move from core laboratory services underscores the challenges in filling and retaining qualified staff within the laboratory systems. Results suggest different strategies are needed to strengthen laboratory management and leadership programs with a clear focus on laboratory systems and laboratory networks to meet current and future clinical and public health laboratory workforce demands.

## Background

The Field Epidemiology Training Program (FETP) was initiated in 1975 as a competency-based training modeled on the Centers for Disease Control and Prevention (CDC's) Epidemic Intelligence Service (EIS) (1). The program trained epidemiologists in countries outside the USA, filling critical gaps in public health programs and remarkably enhancing global health security by increasing the capacity of national public health programs to rapidly detect and respond to public health emergencies. Significant global health security contributions by the graduates of the FETPs included detection of vaccine-type poliovirus in Dominican Republic and Haiti in 1991, *Escherichia coli* O157:H7 in Germany and measles outbreaks in Thailand among others (1, 2). The FETP became a significant mechanism to strengthen epidemiologic capacities globally and by 2016 ~50 countries had adopted it. During the same period, a recognition of emerging and re-emerging diseases as major public health threats led to the need to better integrate laboratory scientists in applied field epidemiology, outbreak response, and disease surveillance. The adoption of the revised International Health Regulations (IHR) in 2007 (3) further exposed gaps in the workforce critical for surveillance and effective response to public health emergencies which requires both epidemiologists and laboratorians to extensively work together. Collaborations between epidemiologists and laboratorians in public health emergences highlighted the need to have joint competency-based trainings, justifying adaptations of the FETP (4). As a result, the first Field Epidemiology and Laboratory Training Program (FELTP) was launched in Kenya in 2004. Laboratory scientists from the Ministry of Health were enrolled in the 2-year program. The FELTP curriculum added a unique laboratory track (L-Track) in addition to epidemiology, disease surveillance, and outbreak responses modules taken by all fellows. Laboratory fellows also took elective modules in laboratory management, quality systems, and diagnostic procedures, and had assignments to laboratory-based field locations (5). The strategic goal was that epidemiologists trained alongside laboratorians would gain mutual understanding of each other's disciplines and strengthen public health surveillance and response.

**Figure 1:**
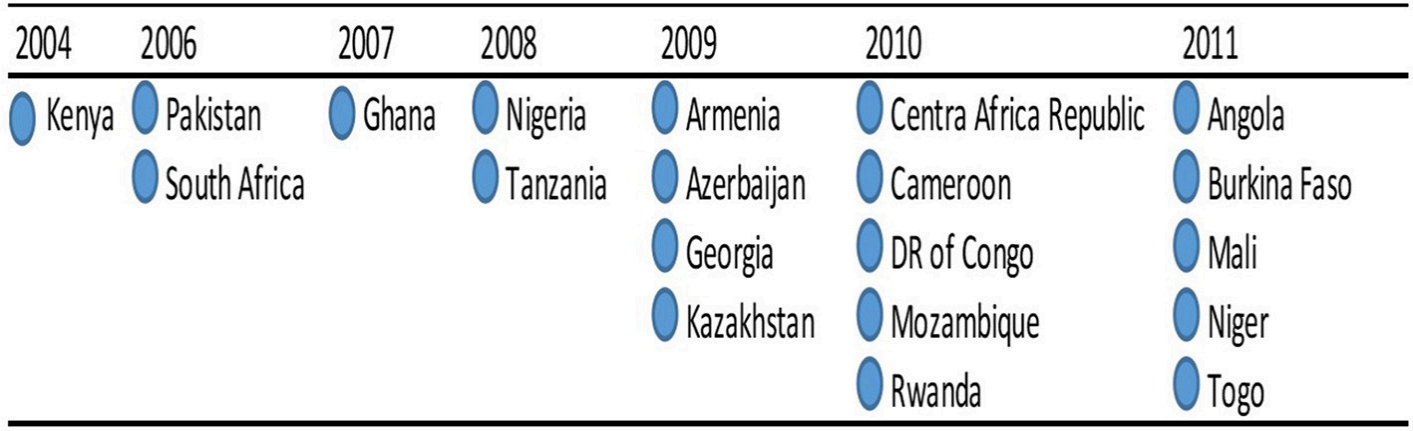
Field Epidemiology & Laboratory Training Programs (FELTP) L-Track Implementation Timeline shows the implementation of Field Epidemiology & Laboratory Training Programs (FELTP) L-Track from 2004 to 2011, a total of 20 countries had either a national or regional FELTP program.

The FELTP laboratory fellows developed competencies through a range of field epidemiology and laboratory investigations of disease outbreaks. Between 2004 and 2005, both epidemiology and laboratory fellows in the Kenya FELTP participated in outbreak responses to aflatoxicosis, brucellosis, and cholera among others (5–7). In Tanzania, FELTP fellows jointly investigated and responded to cholera, anthrax and avian and H1N1 influenza outbreaks among others (8). In addition, FELTP graduates initiated and supported trainings for courses on disease surveillance critical in programs such as Integrated Disease Surveillance and Response (IDSR); further giving the FELTP core impetus within the ministries of health (9, 10). The apparent success of the Kenya FELTP led to establishment of similar programs across Africa and Central Asia by adding the L-Track to existing FETPs. By 2011, 20 countries had FELTP programs (Figure 1), although some were implemented as regional programs e. g., Armenia, Azerbaijan, Ukraine (South Caucus), and Togo, Mali and Burkina Faso (West Africa Region).

Along with the successes however, these FELTP programs have faced major challenges. From the beginning, the curricula were highly variable among the implementing FELTP programs and difficulties arose in integrating the L-Track with the existing FETP curriculum. There were also variabilities in the nature of laboratory specific projects and shortages of qualified mentors and facilities in which the trainees complete their fellowships. No standardized curriculum was developed to cover the core competencies of laboratorians that were being trained together with field epidemiologists. Consequently, FELTP programs tailored the curriculum to their host country needs and capacities. For some programs this meant epidemiologists and laboratory scientists took several laboratory modules with graduates earning a Field Epidemiology degree or certificate. Other programs developed a separate L-Track as a subset of the FELTP with modules for management, policy, laboratory testing for outbreak response and disease surveillance. This was the approach in Kenya with graduates earning a Laboratory Management & Epidemiology degree (5). However, the L-Track of the FELTP lacked clear, systematic, and measurable indicators necessary to comprehensively evaluate and improve the programs (11).

The CDC and many partner FELTP programs have been acutely aware of some of the challenges facing the L-Track despite consensus on the importance of the program. In the evaluation of the FELTP “laboratory component,” workshops reviewing the program and related proposals have been undertaken by programs within CDC's Center for Global Health. One measurable outcome observed from the CDC reviews was that almost a quarter of the FELTP laboratory scientist graduates changed their career to field epidemiologists and thus inadvertently weakening the workforce they were meant to strengthen.

Our assessment combined a review of available documents referencing the status and challenges of the FELTP since its inception and a follow-up questionnaire and discussions that aimed to find out the current state of FELTP programs. With some FELTP programs rolling back L-Track but continuing to enroll laboratory scientists, our aim was to understand the status of the laboratorians in FELTP and profile how the trainees and graduates were fairing within the public health laboratory workforce. The results will inform strategies aimed at strengthening laboratory leadership and give direction to future proposals addressing the public health laboratory workforce.

## Methods

A questionnaire entitled, the FELTP Laboratory Component Survey 2016, was developed to collect information on: (a) year of initial roll-out and current status of the program; (b) challenges or reasons for any roll-back; (c) if program was implemented for single country or at a regional level; (d) total number of FELTP L-Track residents recruited; (e) L-Track minimum qualifications; and (f) positions and institutions of affiliation of the laboratorian residents before recruitment and after graduation. Finally, the participants were given an opportunity to list the major achievements of the FELTP and state what they would change in the FELTP program. The questions were structured as open-ended or with a selection of answers based on the standard FELTP program.

The survey tool was developed in Monkey Survey (www.surveymonkey.com). Questions were designed after consultations on study designs and aim of the survey with all the co-authors. An initial questionnaire was developed and shared with 4 FELTP Resident Advisors (RA) in Georgia, Kazakhstan, Tanzania and Cameroon for validation. Follow-up phone calls and written feedback were used to make changes to the questionnaire before it was sent out to all Resident Advisors (RA) or FELTP point of contacts in FELTP programs. Countries also requested to share records on the training backgrounds, employment histories or any relevant information, in any format they had to track FELTP residents. Data was analyzed both as qualitatively and quantitatively and presented as individual FELTP country profiles. Follow-up phone call and email clarifications were done with FELTP RAs or points of contact. The CDC human subjects research office judged that the survey constituted routine public health activities and therefore did not involve human subjects research.

## Results

### Country response and program status

**Figure 2:**
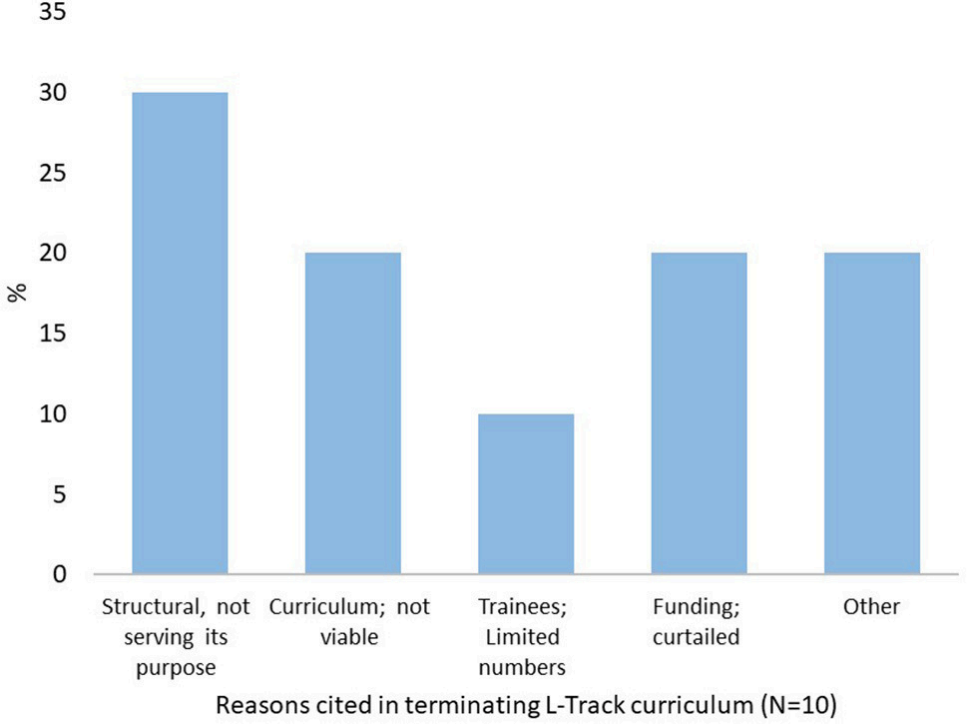
Challenges Identified in Implementing L-Track Curriculum. The main challenges facing the FELTP L-Track implementation as cited by FELTP 10 respondents. Majority stated that FELTP L-Track had structural challenges due to lack of clarity on its purpose. Other reasons included curriculum not viable, limited applicants and funding constrains.

Out of questionnaires sent to FELTP directors in 13 FELTP programs, responses were received from Kenya, Ghana, Nigeria, Tanzania, Armenia/Azerbaijan/Georgia, Kazakhstan, Cameroon, Mozambique, Rwanda, and Mali as of August 2016, representing both national and regional programs. Very limited information was received from Mali, with the respondent indicating the country had residents trained previously in the regional program situated in Burkina Faso in 2011. In 2016, Mali started their own single country program. Since no specific numbers were received, the country's input were only included in Figure 2 below showing challenges with L-Track. From the remaining nine who submitted responses, only Tanzania and Kenya fully completed the questionnaire, including the detailed records of employment histories of all FELTP laboratorian enrollees and graduates. As five FELTP programs had rolled back FELTP programs at the time of the survey, data was only compared between FELTP programs where the specific questions had complete responses.

Nigeria represented the largest number of L-Track residents (total of 72) in FELTP and continues to offer the separate L-Track module (Table 1, below). Kenya, Ghana, and Tanzania had 40 and 38 and 37 L-Track residents respectively. Tanzania, another country with a large number of L-Track residents, had a high graduation rate at 73%. Mozambique, with a smaller cohort, also had a high graduation rate at 73%. Four FELTP programs, including Kenya which first implemented the L-Track module, have rolled back this program since 2014. Kazakhstan initiated the L-Track in 2012 but the program was halted in 2016. Cameroon and Mozambique also rolled back their separate L-Track in 2015 and 2016, respectively. Nigeria, Rwanda, and Tanzania have kept the original program with both Epi and L-Track components. Despite the L-Track roll back, there were no changes in eligibility and all continue to enroll laboratorians in addition to epidemiologists, medical professionals, veterinarians (where applicable) and other scientists who receive the same trainings that includes ~20% laboratory-related topics.

**Table 1:** Status for FELTP Programs (2016).

**Country (sourcing regions)**	**FELTP initiated**	**Laboratory track degree awarded**	**Number of cohorts enrolled**	**Total no. of “L” track FELTP enrolled**	**Total no. of “L” track FELTP enrolled to date (overall graduation rate%)**	**Lab track module (roll back)**
Kenya (Tanzania, South Sudan, Ghana, Uganda)	2004	MSc (Lab Management and Epi)	10	40	20 (50%)	No (2010)
Ghana	2007	MPhil in Field Epi	9	38	23 (61%)	Yes
Tanzania	2008	MSc (Applied Epi and Lab Management)	8	37	27 (73%)	Yes
Nigeria	2008	MPH (Lab Management & Epi)	8	72	31 (43%)	Yes
Georgia (Armenia, Azerbaijan, Ukraine)	2009	Certificate (Non Academic)	n/a	n/a	n/a	Yes
Rwanda	2010	MSc (Applied Epi & Lab Management)	3	9	6 (67%)	Yes
Mozambique	2010	MSc (Applied Epi & Lab Management)	3	15	11 (73%)	No (2016)
Cameroon (DRC, CAR)	2011	MSc (Field Epi & Lab Management)	n/a	n/a	n/a	No (2015)
Mali (Togo, Burkina Faso, Niger)	2008	MPH (Field Epi and Lab Management)	n/a	n/a	n/a	No info
Kazakhstan (FELTP)	2012	Certificate (Non Academic)	2	6	4 (67%)	No 2016

*The initiation, and overall implementation of the FELTP L-Track program. All programs in Africa award a Master degree or its equivalent while programs in Caucuses are non-academic certificate programs. Data on approximate residents trained and status of the L-Track is also shown*.

Fellows in eastern Africa graduated with a Master of Science degree in Laboratory Management and Epidemiology while the western African programs awarded Master of Philosophy or Masters in Public Health in Field or Applied Epidemiology and Laboratory Management (Table 1). The Kazakhstan and Central Asian programs are non-degree. Data regarding numbers of L-Track residents or graduates were not available for regional programs in Cameroon, Mali and Georgia.

### Basic entry qualifications

For all FELTP programs, the basic academic qualification for fellows entering the L-Track was a bachelor's degree in laboratory sciences (or equivalent) as shown in Table 2 below. In Ghana there was an indication those with diploma in laboratory sciences also qualify but the diploma training is a 3-year course in clinical laboratory post high school and thus would be functionally equivalent to a bachelor's degree in laboratory sciences. Those with Medical or Veterinary degrees or Masters degrees or higher are eligible in all implementing FELTP programs. However, there are differences in the work experience required to qualify for FELTP enrollment. This ranged from a high of 3–5 years in clinical or public health laboratory work experience (Kazakhstan and Tanzania) to no work experience necessary in Nigeria.

**Table 2:** Academic and work experience requirements for FELTP trainees.

**Countries**	**Ghana**	**Kazakhstan**	**Kenya**	**Mozambique**	**Nigeria**	**Rwanda**	**Tanzania**
Basic academic qualifications	BSc Lab Sciences, Diploma Lab Science	BSc Lab Sciences	BSc Lab Sciences	BSc Lab Sciences	BSc Lab Sciences	BSc Lab Sciences	BSc Lab Sciences
Work experience	1–2 years in Clinical/Public Health Laboratory	3–5 years in Clinical/Public Health Laboratory	At least 2 years in Clinical/Public Health Laboratory	1–2 years in Clinical/Public Health Laboratory	No work experience necessary	1–2 years in Clinical/Public Health Laboratory	3–5 years in Clinical/Public Health Laboratory

*The basic qualifications for admission to the FELTP L-Track Program both in academics and work experience. All require a degree in or Diploma in Laboratory sciences, Tanzania and Kazakhstan require at least 3 years work experience while no work experience in required in the Nigeria program*.

### Challenges in L-Track implementation

Several respondents cited factors they considered major challenges which are either ongoing or contributed to the decision to roll back the separate L-Track curriculum (Figure 2). Respondents cited a lack of a standardized curriculum for the laboratory module, describing it as “curriculum not viable” or “structural issues and is not serving its purpose” while others cited shortage of laboratorian applicants. Another recurring factor were budget restrictions and lack of sustainability options. A combination of some of these reasons and budget cuts affecting the whole FELTP program also resulted to some scaling back on the separate laboratory module. Most of the budgetary issues were linked to reductions in funding for programs such as Presidential Emergency Plan for AIDS Relief (PEPFAR) and Presidential Malaria Initiative (PMI), and other related international aid programs for public health.

### Source of L-Track residents in FELTP

**Figure 3:**
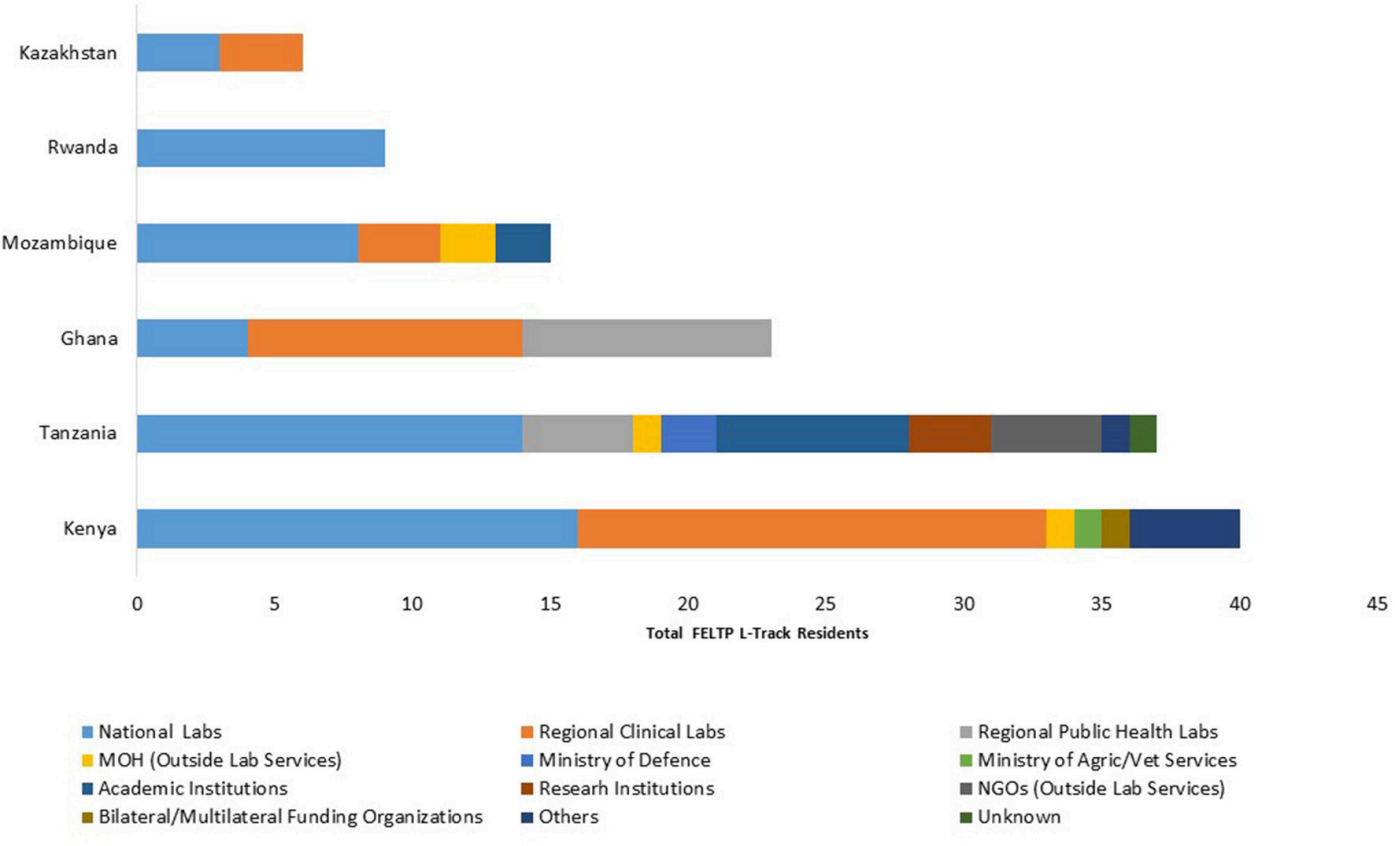
Source Institutions for FELTP Laboratory Residents. The institutional recruitment sources of FELTP L-Track trainees. Majority of recruitments were sourced from national and regional public health or clinical laboratories. A small number were from Ministry of Agriculture, nongovernmental organization and academic institutions.

Data from the FELTP programs with complete information indicated the bulk of residents were recruited from national public health laboratories or from the regional public health or clinical laboratories. Ghana had the largest proportion from regional public health laboratories and clinical laboratories (Figure 3). Kenya and Tanzania had the widest range of institutions contributing to FELTP L-Track residents which in addition to Ministry of Health included research institutes, Ministry of Agriculture and Livestock, Ministry of Defense, universities and non-governmental organizations outside of the traditional public health laboratory network. In contrast, all the FELTP L-Track residents in Rwanda came from the national reference laboratory. In Kazakhstan, residents were equally divided from the national reference laboratory and regional laboratories.

### FELTP L-Track residents in six FELTP programs

**Figure 4:**
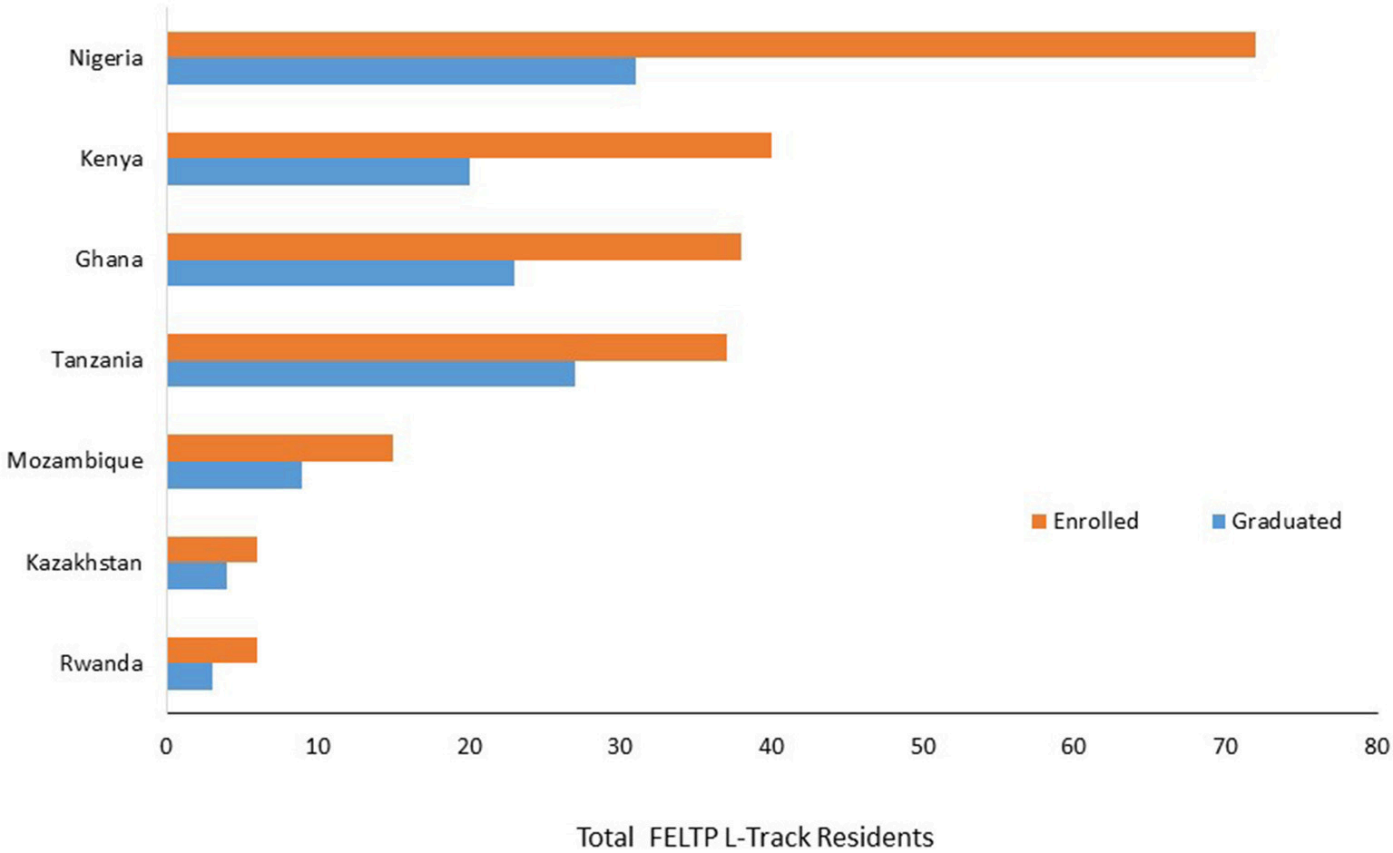
Cumulative Enrollment and Graduates of FELTP L-Track in 7 Countries. The cumulative number of laboratory residents enrolled in FELTP from beginning of each program up to the time of survey in April 2016. Nigeria has the highest cohort and continue to build their number of trained FELTP L Track residents.

FELTP programs continue to build their capacity by steadily enrolling laboratorians in their FELTP. Nigeria has the largest number of trainees (101). The graduation rate is low in Nigeria at 31%, but this is because of an upswing in recruitment in the last four cohorts. Kenya continues to enroll laboratorians in their FELTP, after rolling back the L-Track as does Mozambique and Ghana. Tanzania had more FELTP L-Track graduates than the trainees in the pipeline as shown in Figure 4.

#### Where are they now?

**Figure 5:**
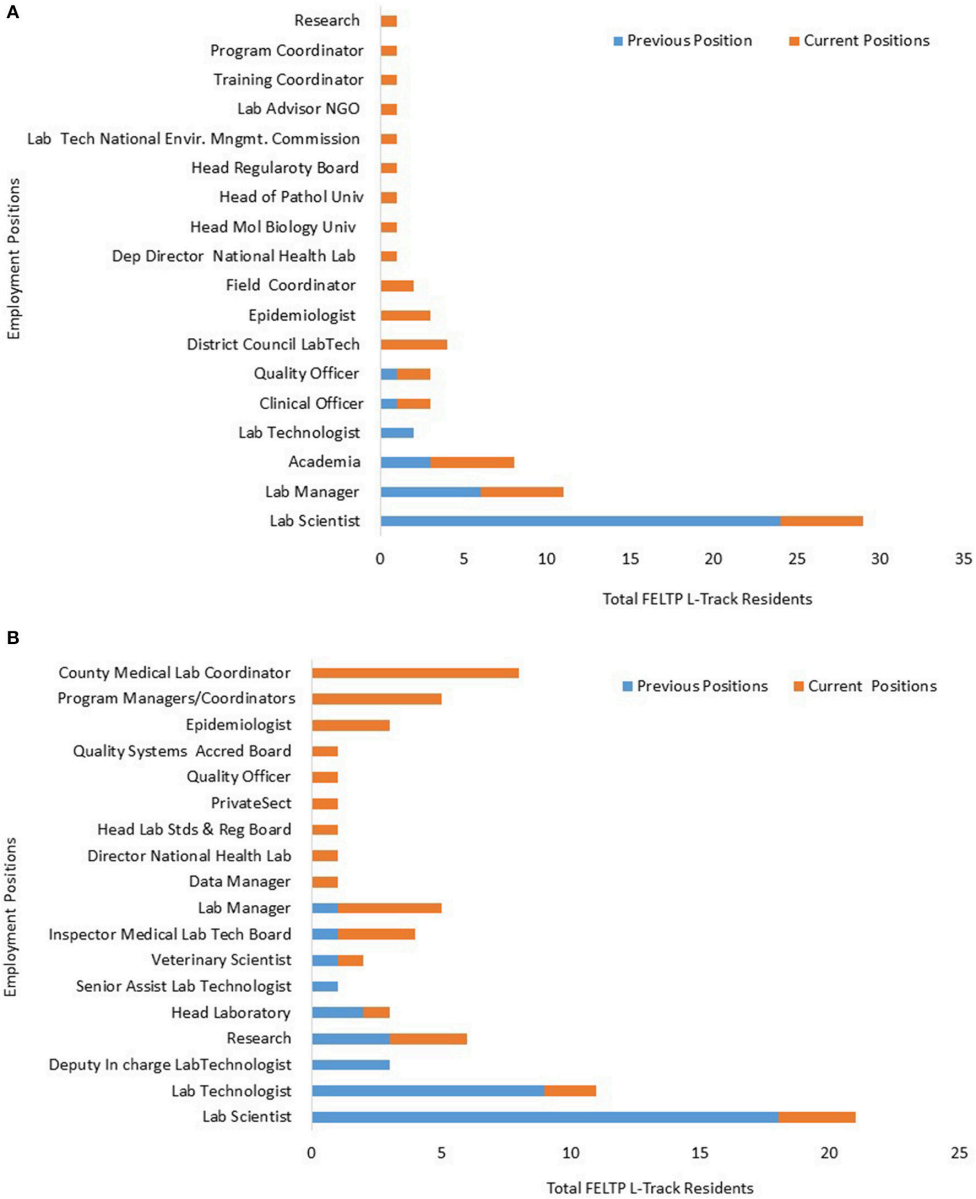
Tanzania FELTP lab resident positions. **(A)** The employment position categories for FELTP residents prior and post enrollment in Tanzania. Majority of residents were laboratory scientists before joining FELTP but there was a big diversification of areas where residents were employed post enrollment. **(B)** Kenya FELTP Laboratory Residents Positions. The employment position categories for FELTP residents prior and post enrollment in Kenya. Majority of residents were laboratory scientists before joining FELTP but there was a big diversification of areas where residents were employed post enrollment, a majority becoming Coordinators.

A major concern for the FELTP L-Track has been the career trajectory of the graduates. Unfortunately, only Kenya and Tanzania provided the detailed data on where their residents were before and after enrollment into FELTP. Out of 40 so far enrolled in Kenya, complete data was provided for 39 FELTP Laboratory residents while Tanzania provided complete data for 37 residents. Prior to enrollment, the majority of residents in both Kenya and Tanzania were laboratory scientist working in either the national public health laboratories or in district or provincial hospital clinical laboratories. Areas of employment of residents showed diverse positions as seen in Table 3. For the Tanzania cohorts, there were ~6 general categories of the positions prior to enrollment with the largest group comprising 65% laboratory scientists. This number of categories increased to 17 post-enrollment (Figure 5A). Similarly, in Kenya there were eight overall categories with the laboratory scientists making about 46% of the total. Conversely, Kenya post enrollment categories were double the pre-enrollment numbers at 16 (Figure 5B). It is unclear if the residents changed positions because of enrollment into the FELTP as some of them were yet to graduate.

**Table 3:** Institutional mobility of FELTP residents in Kenya and Tanzania.

**Resident positions & institutional mobility**
**Propotions**	**Kenya** ***n*** = **39**	**Tanzania** ***n*** = **37**
**Mobility**	**%**	**%**
At original institutions	62	30
Moved institutions	38	70
**PROMOTIONS POST FELTP ENROLLMENT**		
Promotion mobility	87	73
Same position	18	27

*The positions of residents in both Kenya and Tanzania, before and after enrollement into FELTP. There was a high movement between institutions in the Kenya residents but in both countries, residents reported moving to a higher position after enrollement*.

While the Kenya and Tanzania cohorts exhibited this change in positions, analysis indicated 62% of the Kenyan cohort moved to a different institution while 70% in Tanzania were in the same institution post enrollment but largely within the public sector. However, there was a high level of upward mobility with both cohorts indicating an upward change in position at 87 and 73% in Kenya and Tanzania respectively as shown in Table 3 below.

### FELTP placement post-training in Kenya and Tanzania

From the overall positions of the residents post-FELTP enrollment, a classification was made on possible areas of focus to assess the proportion of those who are still working directly in the laboratory services. Those considered to be working directly in the clinical and public health laboratory services included laboratory directors or deputies, laboratory managers, heads of laboratory units, quality officers, blood safety, scientists, and technologists in laboratories including in academics and research. Those considered not working directly in laboratory services included residents in program managements, field and training coordinators, local government management positions (counties, district councils), epidemiologists, FELTP Advisors, inspectors and bureau of standards managers. Others not considered directly in laboratory services were in NGOs and the private sector. Approximately 56% of residents in Kenya work in areas considered outside laboratory services while the corresponding number is much lower in Tanzania at 43%. A significant proportion of FELTP graduates in Kenya are working as laboratory coordinators with county governments or in international organizations including WHO, CDC, and African Field Epidemiology Network (AFENET). A small but significant number of L-Track were international students and data show they have also progressed to be: the head of national laboratory in Tanzania, the head of laboratories for the Ministry of Defense, and the laboratory inspector both in South Sudan, and program officer for laboratory quality assurance in the Uganda Central Public Health Laboratory. One graduate has joined the private sector while there was loss to follow up for another graduate. In Tanzania, one of the graduates was a clinical officer and rejoined the source institution (military hospital) in Zambia.

## Responses from open-ended questions

At the end of the questionnaire, respondents listed recommendations for changes in the FELTP L-Track. Recommendations included:

- Dropping the L-Track and creating a separate laboratory leadership program but with clear intersections with the FETP;
- Revising the L-Track curriculum and aligning it with public health laboratory needs and also improving the evaluation bench marks;
- Emphasizing laboratory data analyses and introducing advanced testing (such as genome sequencing);
- The L-Track would benefit greatly with technical support from CDC headquarters support similar to that given to FETP. The Resident Advisors noted that FETP support is highly structured and effective but this is not the case for the L-Track which is highly variable based on implementing FELTP program.

## Discussion and conclusion

The introduction of FELTP, especially in Africa, has seen a paradigm shift in laboratory training in the era of emerging and re-emerging diseases, disease detection and integrated disease surveillance and an important enhancement in global health security by contributing to more rapid detection and control of public health emergencies. (12–14). The FELTP offers laboratorian skills that were hitherto neglected by the traditional academic trainings which offered little in laboratory management or competencies in integrated epidemiology, laboratory surveillance, confirmatory testing in disease outbreaks and data analyses (15). Despite the demand for skilled and competent laboratorians in public health systems, the lack of standardized curriculum or long-term strategies threaten the sustenance of the separate L-Track as is evident from the roll back from many FELTP programs. In response, countries are finding the middle ground for FELTP by continuing to recruit laboratorians into the training where laboratory related modules are covered in the same scope with epidemiologists, veterinarians and any other trainees. This is the case with other FET(L)P programs in Kenya, Cameroon, and Central Asia and South Caucus which even without the L-Track enroll laboratory scientists for training in applied or field epidemiology (16). Nigeria, Rwanda and Tanzania however continue to implement and expand the L-Track recruitments to the respective FELTP programs. Reports form AFENET also show Angola continues to enroll L-Track and the 2 cohorts of 20 residents comprised of 12 Epi and 8 L-Track (15).

The challenges facing the L-Track FELTP identified here were listed in previous evaluations done by CDC. The program has experienced structural problems some of which may be inherent in the respective countries laboratory systems. For example, majority of recruits to the L-Track within the FELTP were from either the national reference laboratories or the regional clinical laboratories which in themselves have significant challenges in their operation and management systems. It is worth noting that prior to 2004 when the first FELTP cohort was initiated in Kenya, medical laboratories in many developing countries were severally neglected with low standards, undefined or outdated policies and limited national government's funding (17). The L-Track has therefore operated in an era where national laboratory systems were starting to address systemic challenges including legal frameworks, mandates and standards for medical laboratories (18). The lack of clarity at the national laboratory systems level and sometimes absence of defined public health laboratories ultimately may contribute to some of the structural challenges in both the curriculum and career development. Some of these were highlighted in a previous review where lack of sufficient mentors, key capacities in laboratory settings, lack of infrastructure and supplies were identified as challenges to FELTP among others (CDC; internal presentation). In addition, with the bulk of funding for FELTP tied to clinical laboratory testing, alignment to the core competencies of field laboratory and epidemiology was difficult. As donor funds have decreased, FELTP has seen significant budget cuts disrupting programs as they search for alternative financing. Some FELTP programs such as in Nigeria however continue to expand their L-Track program representing a unique program with joint training for epidemiologists, public health laboratory scientists, and veterinary field epidemiologists.

Several FELTP programs indicated the curriculum was “not viable.” Further discussions revealed programs were still debating on what the L-Track aim was considering the tendency of turning laboratorians into epidemiologists or program managers. Indeed, our data indicate over 50% of the FELTP residents in Kenya are working as epidemiologists, program managers or coordinators while in Tanzania 43% are working as epidemiologist or program managers/coordinators. As the data shows most of this was through career changes and promotions, an indication of the ambiguity of the program in relation to the respective workforce development especially in strengthening public health systems, laboratory response to outbreaks and disease surveillance. Despite this limitation, the data suggests the FELTP graduates are in demand especially in program coordination and management. At least four graduates are working as FELTP RA or advisors for CDC, or AFENET while others have joined international organizations including WHO.

A growing number of residents have taken positions as county medical laboratory coordinators in Kenya or district council laboratory scientists in Tanzania. The devolutions of national governments to a more decentralized system is opening opportunities at local government levels for laboratory coordinators and manager to provide oversight on services, budgets, personnel, infrastructure, and trainings. These are senior positions in the current health services delivery system as they are members of county/regional Senior Health Management Teams (SHMTs). Their skills in clinical laboratories and competencies in field epidemiology and laboratory management afford them unique opportunities to oversee both diagnostic and surveillance services. However, while this may be a priority with the country's national health system, and an affirmation of the program's success, it is a drain to the public health laboratory systems that were targeted in the initial FELTP programs, reducing the number of highly qualified staff within laboratories. There are other laboratory training programs aiming at building competencies in laboratory quality management, or management and leadership, but these are mainly short term, specific program-oriented and often times aim for facility-based improvements, rather than national systems strengthening (19, 20).

A downside to this survey was the limited data available for analysis as most FELTP programs failed to complete the questionnaire in its entirety. There is also evidence that databases one on the positions of fellows before joining FELTP and post-graduation and career paths within the health sector is not always available. Individual country data and reports on career progression of their residents over time would inform on the significance of the programs but there are significant challenges in getting complete data. This information is important in the development of strategies to address the core needs within public health laboratory workforces to fill the demands in clinical laboratories and integrated disease surveillance. The recent Ebola outbreak in parts of West Africa has increased the demand for laboratorians competent in disease surveillance, management of outbreaks and overall leadership skills (13, 21). Such skills remain a significant gap in laboratories workforce despite the implementation of FELTP program.

In conclusion, there is still a lack of clarity in both structure and systems in countries regarding the L-Track of the FELTP. Confusion still lingers on graduates' career paths and their advancements within the respective ministries. The majority of responders indicated it would be preferable to have a more focused field laboratory management program with clear intersection and overlap with epidemiologists. This could be achieved through an independent in-service laboratory professional training program with strong links to FETP or a more robust autonomous laboratory training track within the umbrella of FELTP. A concerted effort should also focus on host countries reviewing their laboratory workforce policies to include FELTP and other areas in laboratorian career development programs that are aligned and reflect the current demand for skilled and competent laboratory scientists able to strengthen public and clinical laboratory systems.

## Availability of data and materials

The questionnaire and raw responses generated and/or analyzed during the current study are not publicly available as they are held as program reports but would be availed from the corresponding author on reasonable request. The summarized reports obtained are however inclusive in whole of all data obtained.

## Ethics statement

The CDC human subjects research office judged that the survey constituted routine public activities and therefore did not involve human subjects research. All respondents were contacted by phone and email and they responded to voluntarily participate in the Survey Monkey answers. All authors submitted a written consent to the contents and publication of this manuscript in its entirety. The results and draft manuscript was shared with all the participants as feedback. The manuscript was reviewed and cleared for publication by Centers for Disease Control and Prevention.

## Author contributions

WG is the lead author and is accountable for data analyses and all other aspects of this manuscript. AH, MR, JM, LP, and AAA were key in the study design and drafting of the questionnaire. TG, AA, and DM contributed to the in depth data summary data of resident's positions used in this study. All authors contributed to final discussion in the manuscript and all approved the final version for submission to scientific publication.

### Conflict of interest statement

The authors declare that the research was conducted in the absence of any commercial or financial relationships that could be construed as a potential conflict of interest.

## Abbreviations

AFENET: African Field Epidemiology Network
CDC: Centers for Disease Control and Prevention
FETP: Field Epidemiology Training Program
FELTP: Field Epidemiology and Laboratory Training Program
IDR: Integrated Disease Surveillance and Response
IHR: International Health Regulations
L-Track: Laboratory Track
PEPFAR: Presidential Emergency Plan for AIDS Relief
PMI: Presidential Malaria Initiative
RA: Resident Advisors
SHMTs: Senior Health Management Teams
WHO: World Health Organization.

## References

[1] White ME McDonnell SM Werker DH Cardenas VM Thacker SB. Partnerships in international applied epidemiology training and service, 1975-2001. Am J Epidemiol. (2001) 154:993–99. 10.1093/aje/154.11.993 11724714

[2] Kew O Morris-Glasgow V Landaverde M Burns C Shaw J Garib Z. Outbreak of poliomyelitis in Hispaniola associated with circulating type 1 vaccine-derived poliovirus. Science (2002) 296:356–9. 10.1126/science.1068284 11896235

[3] WHO International Health Regulations. WHO Library Cataloguing-in-Publication Data, 3rd ed (2005).

[4] Nsubuga P Johnson K Tetteh C Oundo J Weathers A Vaughan J. Field Epidemiology and Laboratory Training Programs in sub-Saharan Africa from 2004 to 2010: need, the process, and prospects. Pan Afr Med J. (2011) 10:24. 10.11604/pamj.2011.10.24.1271 22187606 PMC3224071

[5] Kariuki Njenga M Traicoff D Tetteh C Likimani S Oundo J Breiman R. Laboratory epidemiologist: skilled partner in field epidemiology and disease surveillance in Kenya. J Public Health Policy (2008) 29:149–64. 10.1057/jphp.2008.3 18523470

[6] Nguku P Mosha F Prentice E Galgalo T Olayinka A Nsubuga P. Field epidemiology and laboratory training programs have been in Africa for 10 years, what is their effect on laboratory-based surveillance? Reflections from a panel at the African Society of Laboratory Medicine December 2014 Cape Town meeting. Pan Afr Med J. (2015) 20:451. 10.11604/pamj.2015.20.451.6787 26309482 PMC4537889

[7] Shuaib F Gunnala R Musa EO Mahoney FJ Oguntimehin O Nguku PM. Ebola virus disease outbreak - Nigeria, July-September 2014. MMWR Morb Mortal Wkly Rep. (2014) 63:867–72. 25275332 PMC4584877

[8] Mmbuji P Mukanga D Mghamba J Ahly M Mosha F Azima S. The tanzania field epidemiology and laboratory training program: building and transforming the public health workforce. Pan Afr Med J. (2011) 10 (Suppl. 1):9. 22359697 PMC3266678

[9] Becker KM Ohuabunwo C Ndjakani Y Nguku P Nsubuga P Mukanga D. Field Epidemiology and Laboratory Training Programs in West Africa as a model for sustainable partnerships in animal and human health. J Am Vet Med Assoc. (2012) 241:572–9. 10.2460/javma.241.5.572 22916854

[10] Mukanga D Namusisi O Gitta SN Pariyo G Tshimanga M Weaver A. Field epidemiology training programmes in Africa - where are the graduates? Hum Resour Health (2010) 8:18. 10.1186/1478-4491-8-18 20696029 PMC2927471

[11] Rush T. Disease surveillance system evaluation as a model for improved integration and standardization of the laboratory component in the Field Epidemiology and Laboratory Training Program (FELTP) curriculum worldwide. J Public Health Policy (2012) 33:390–400. 10.1057/jphp.2012.35 22971950 PMC5716808

[12] Gitta SN Mukanga D Babirye R Dahlke M Tshimanga M Nsubuga P. The African Field Epidemiology Network–networking for effective field epidemiology capacity building and service delivery. Pan Afr Med J. (2011) 10 (Suppl. 1):3. 22359691 PMC3266672

[13] Lubogo M Donewell B Godbless L Shabani S Maeda J Temba HEbola virus disease outbreak; the role of field epidemiology training programme in the fight against the epidemic, Liberia, 2014. Pan Afr Med J (2015) 22 (Suppl. 1):5 10.11694/pamj.supp.2015.22.1.6053 PMC4709128 26779298

[14] Ntahobakurira I Antara S Galgalo TB Kakoma JB Karema C Nyatanyi T. The rwanda field epidemiology and laboratory training program: training skilled disease detectives. Pan Afr Med J. (2011) 10 (Suppl. 1):7. 22359695 PMC3266676

[15] AFENET Annua Report. Available online at: http://www.afenet.net/index.php/resources/report/191-annual-report-2014.

[16] Masanza MM Nqobile N Mukanga D Gitta SN. Laboratory capacity building for the International Health Regulations (IHR[2005]) in resource-poor countries: the experience of the African Field Epidemiology Network (AFENET). BMC Public Health (2010) 10 (Suppl. 1):S8. 10.1186/1471-2458-10-S1-S8 21143830 PMC3005580

[17] Petti CA Polage CR Quinn TC Ronald AR Sande MA. Laboratory medicine in Africa: a barrier to effective health care. Clin Infect Dis. (2006) 42:377–82. 10.1086/499363 16392084

[18] Nkengasong JN Mesele T Orloff S Kebede Y Fonjungo PN Timperi R. Critical role of developing national strategic plans as a guide to strengthen laboratory health systems in resource-poor settings. Am J Clin Pathol. (2009) 131:852–57. 10.1309/AJCPC51BLOBBPAKC 19461093

[19] Perrone LA Voeurng V Sek S Song S Vong N Tous C. Implementation research: a mentoring programme to improve laboratory quality in Cambodia. Bull World Health Organ. (2016) 94:743–51. 10.2471/BLT.15.163824 27843164 PMC5043202

[20] Yao K McKinney B Murphy A Rotz P Wafula W Sendagire H. Improving quality management systems of laboratories in developing countries: an innovative training approach to accelerate laboratory accreditation. Am J Clin Pathol. (2010) 134:401–09. 10.1309/AJCPNBBL53FWUIQJ 20716796

[21] Preacely N Nsubuga P. Influenza preparedness and response: involvement of African Field Epidemiology and Laboratory Training Programs, 2009. Pan Afr Med J. (2011) 10:11. 10.11604/pamj.2011.10.11.1139 22187593 PMC3282936

